# FACTORS INFLUENCING DEMENTIA DIAGNOSIS AWARENESS AMONG RACIALLY/ETHNICALLY DIVERSE OLDER ADULTS

**DOI:** 10.1093/geroni/igae098.0045

**Published:** 2024-12-31

**Authors:** Maria Roche-Dean, Sol Baik, Noah Webster, Heehyul Moon, Laura Zahodne

**Affiliations:** University of Kansas, Kansas City, Kansas, United States; University of Virginia, Charlottesville, Virginia, United States; University of Michigan, Ann Arbor, Michigan, United States; University of Louisville, Louisville, Kentucky, United States; University of Michigan, Ann Arbor, Michigan, United States

## Abstract

Black and Hispanic adults arrive in later life with a greater risk for Alzheimer’s Disease and Related Dementias (ADRD). Untimely dementia diagnoses continue to be a challenge in non-Hispanic Black and Hispanic-identified older individuals, delaying symptom management and access to health services. In this study, we examine whether social factors are associated with awareness of dementia diagnosis among older adults with probable dementia. Data were from the National Health and Aging Trends Study round 1, a nationally representative sample of Medicare beneficiaries aged 65 and older assessed in 2011. Those selected for analysis met algorithmic criteria for probable dementia (N=561). Logistic regression was performed to assess the influence of marital status, social network size, and number of helpers on the odds of reporting a dementia diagnosis, controlling for demographics. In the whole sample, younger individuals and women had greater odds of dementia diagnosis awareness. Race/ethnicity, marital status, the number of helpers, and social network size were not associated with dementia diagnosis awareness. Analyses stratified by race/ethnicity indicated that being married was only associated with greater odds of dementia diagnosis awareness among Black participants. The lack of association between social network, number of helpers, and dementia awareness could reflect limitations on how probable dementia and dementia awareness were operationalized in this dataset. Furthermore, marital support may be important for timely diagnosis for Black individuals with dementia.

